# Effects of the Football+ and 11+ Warm‐Up Programs on Motor Performance Among Amateur Female Players: A 12‐Week Intervention With a 10‐Week Follow‐Up

**DOI:** 10.1002/ejsc.70287

**Published:** 2026-11-03

**Authors:** Mojtaba Asgari, Martin Hägglund, Marcus Schmidt, Benedikt Terschluse, Maximilian Sueck, Thomas Jaitner

**Affiliations:** ^1^ Institute for Sport and Sport Science TU Dortmund University Dortmund Germany; ^2^ Department of Health Medicine and Caring Sciences Unit of Physiotherapy Linköping University Linköping Sweden

**Keywords:** football, injury prevention, physical ability, technical skills, warm‐up

## Abstract

This three‐armed cluster‐allocated interventional study aimed to investigate the effects of a 12‐week Football+ and 11+ warm‐up programs on motor performance and the retention of potential adaptations following a 10‐week no‐intervention period. Five amateur female football teams voluntarily participated, completed the introductory sessions, and were team‐allocated to the 11+ (*n* = 20, 23.6 ± 3.9 years, 1.69 ± 0.07 m, 65.4 ± 8.5 kg), the Football+ (*n* = 38, 26.5 ± 5.7 years, 1.68 = ± 0.09 m, 63.4 ± 7.5 kg), and Control group (*n* = 30, 22.53 ± 3.2 years, 1.66 ± 0.06 m, 64.2 ± 7.8 kg). The experimental groups completed a 12‐week supervised intervention twice weekly, whereas the control group continued its warm‐up routine. Motor performance was assessed pre‐ and post‐intervention using 20‐m sprint, countermovement jump (CMJ), Illinois agility (IA), and dribbling speed (DS) tests. A follow‐up assessment was conducted after a 10‐week no‐intervention period to address retention of the potential improvements. Statistical significance was set at *α* < 0.05. Linear mixed‐effects model analysis revealed a significant time × group interactions for 20‐m sprint, CMJ, IA, and DS (all *p* = 0.001–0.013). The Football+ group demonstrated the greatest performance gains across all tests, with sprint time improving by Δ − 0.13 s, CMJ height by Δ + 2.5 cm, agility time by Δ − 0.91 s, and dribbling time by Δ − 0.29 s. The gains obtained did not persist after 10 weeks without intervention. A 12‐week supervised training with the Football+ enhanced motor performance measures among amateur female players compared with the 11+ and traditional warm‐ups. The absence of sustained effects at follow‐up indicates that performance gains are dependent on continued use of the intervention.

**Trial Registration:**

DRKS00038406

## Highlights

- The Football+ warm‐up program led to greater improvements in sprinting, agility, jumping, and dribbling performance than the 11+ or traditional warm‐ups.
- Performance gains disappeared after 10 weeks without the program, showing the need for continued use.
- Because it is football‐specific, requires no equipment, and takes the same time as common warm‐ups, coaches can adopt the Football+ without changing their training structure.

## 1 Introduction

Women's football has undergone remarkable growth over the past 2 decades. While fewer than 600,000 female players were registered worldwide in the late 1990s, today more than 30 million women play football across 176 countries, representing approximately 10% of the global football population (Del Coso et al. 2018; Geertsema et al. 2021). Despite this expansion, scientific investigations focusing on women's football remain comparatively limited, rendering it an under‐researched athletic population. Injury surveillance studies conducted in women's football leagues across Europe, the Americas, and Africa emphasize that the incidences and severity of injuries among female players are as high as those observed in their male counterparts (Babwah 2014; Ekstrand et al. 2011; Faude et al. 2005; Hartmut et al. 2010; Horan et al. 2022; Ibikunle et al. 2019; Jacobson and Tegner 2007; Larruskain et al. 2018; Robles‐Palazón et al. 2022; Tegnander et al. 2008).

Numerous randomized controlled trials (RCTs) have demonstrated the efficacy of injury prevention programs (IPPs) in reducing football‐related injuries by 36%–75%, with the FIFA11+ warm up program (the 11+) standing out as the most studied IPP (Emery et al. 2022; Soligard et al. 2008). Research has also shown a strong association between lower injury rates and team success in football (Eirale et al. 2013). Nevertheless, the delivery of the IPPs into the football community and their implementation in practice remains a formidable challenge (Al Attar et al. 2017; O’Brien et al. 2017; Wilke et al. 2018). Convincing coaches, trainers, and players to fully embrace and follow these programs remains difficult, largely due to their limited impact on performance enhancement, malalignment with football‐specific goals, lack of time, and player enjoyment and engagement (Asgari, Nazari, et al. 2022b; O'Brien et al. 2017; Winstanley et al. 2023).

To reflect this issue, a football‐specific warm‐up program, named Football+, was recently developed. By integrating football‐specific exercises with injury prevention routines into a structured warm‐up, the Football+ aims to overcome the aforementioned challenges associated with IPP adoption. Building on existing literature and recommendations to improve the 11+ (Asgari, Alizadeh, et al. 2022a; Asgari, Nazari, et al. 2022b; Bathe et al. 2023; Dalen‐Lorentsen et al. 2024; O'Brien and Finch 2016; O'Brien et al. 2017; O’Brien et al. 2017; Wilke et al. 2018; Winstanley et al. 2023), the Football+ is designed to reduce injury risk while enhancing motor performance. This dual focus is expected to promote greater acceptance and compliance among football associations, and warrant easy implementation with minimal equipment, making it feasible for widespread use across various levels of play.

Initial investigations indicated that the Football+ is superior to both the 11+ and traditional warm‐ups in optimizing acute performance, including sprinting, agility, and dribbling speed with large effect sizes (Asgari et al. 2023). Four months of supervised training with the Football+ has also shown significant improvements in landing quality among female players with and without a history of severe ankle or knee injury (Asgari, Hägglund, et al. 2026b). Further, a recent study has demonstrated significant changes in knee injury risk indicators following 3 months of supervised training with the Football+ and 11+ programs among amateur female players with more consistent and larger effects observed for the Football+ (Asgari, Hägglund, et al. 2026a). However, its effects on motor performance over time remain unexplored. As the Football+ was specifically designed as a dual‐purpose program targeting both performance enhancement and injury risk reduction, evaluating its impact on motor performance outcomes is essential to justify its implementation as a time‐efficient warm‐up strategy in applied football settings. Further, although the effects of the 11+ on motor performance have been investigated in adult male players, evidence regarding its effectiveness in female players is lacking (Asgari, Nazari, et al. 2022b). Given that sex‐related differences influence neuromuscular characteristics and motor performance profiles, examining the performance‐related effects of the 11+ in female players is necessary to provide evidence‐based guidance for coaches and practitioners working in women's football.

Additionally, assessing retention of potential improvements provides practical insight into the durability of adaptations and helps determine whether ongoing exposure is necessary to sustain the benefits of the programs. Such information is especially relevant for IPPs like the 11+ and Football+, which are intended for continuous application throughout the competitive season. Therefore, this study aimed to evaluate the effects of a 12‐week supervised training with the Football+ and 11+ programs on motor performance among amateur female players. We hypothesized that the application of the Football+ would yield superior performance outcomes in female players and to explore whether potential gains persist after a 10‐week no‐intervention period.

## 2 Materials and Methods

### 2.1 Study Design and Ethical Considerations

This registered (DRKS00038406) three‐armed cluster‐allocated non‐randomized trial adhered to the guidelines of the Helsinki Declaration. Participation was voluntary, and all participants provided written informed consent before the study commenced. The ethics committee of TU Dortmund University reviewed and approved the study protocol (GEKTUDO_2022_43).

### 2.2 Participants

G*Power analysis determined a sample of 84 for an expected effect size of *F* = 0.2, *α* error probability of 0.05 and power of 0.90 (*F*‐test family, ANOVA‐within‐between interaction) (Faul et al. 2009; Serdar et al. 2021). Accordingly, a convenience sample of five adult female teams competing in the German amateur leagues (Landesliga) was invited and voluntarily participated in this study. Except for one team who agreed to participate as a control group only, the others were randomly allocated by team to one of two intervention groups, the Football+ (2 teams, *n* = 38) and the 11+ (one team, *n* = 20) or a control group (2 teams, *n* = 30). Of the 94 players initially recruited, 6 were excluded according to the study criteria, resulting in a final sample of 88 players: baseline demographics, exposure levels, and follow‐up availability of the players are presented in table one. Although the final analyses were performed using linear mixed‐effects models to account for team‐level clustering and incomplete follow‐up data, the initial sample size calculation was used to guide participant recruitment and should be interpreted in the context of the cluster‐allocated study design. Details are presented in Table 1.

**TABLE 1:** Baseline demographics, exposure levels, and follow‐up participation.

Variable	Football+ (*n* = 38)	11+ (*n* = 20)	Control (*n* = 30)
Age (years)	26.5 ± 5.7	23.6 ± 3.9	24.53 ± 3.16
Height (m)	1.68 ± 0.09	1.69 ± 0.07	1.66 ± 0.06
Body mass (kg)	63.4 ± 7.5	65.4 ± 8.5	64.2 ± 7.8
Level of play	Amateur league	Amateur league	Amateur league
Weekly training frequency	3 sessions/week (2 with intervention)	3 sessions/week (2 with intervention)	3 sessions/week sessions/week
Match frequency	1 match/week	1 match/week	1 match/week
^a^Follow‐up availability	1 team available (*n* = 26)	1 team available (*n* = 20)	NA

^a^ Refers to the 10‐week no‐intervention period.

### 2.3 Study Criteria

Players eligible for participation were injury‐free at baseline and registered with their respective teams for the 2023‐2024 season. Exclusion criteria included missing more than 10% of intervention sessions, simultaneous participation in other sports activities, absence from any measurement session, or sustaining a severe injury (> 4 weeks absence) during the study period.

### 2.4 Study Preparation and Interventions

Before the study began, the objectives, measurements, and interventions were thoroughly explained to the coaches, trainers, and players. Intervention groups were provided with supplementary materials, video tutorials, posters, and manuals for their specific programs. Close collaboration with the teams was maintained throughout the intervention period to ensure adherence and foster consistent participation.

Both intervention programs consist of three parts, lasting 22–25 minutes in practice and requiring no equipment, but differ in content. Specifically, the Football+ incorporates a greater volume of football‐specific and high‐intensity neuromuscular activities, including multidirectional movements, ball‐oriented coordination tasks, acceleration and deceleration actions, reactive drills, and sport‐specific agility components. It consists of three progressive parts, each targeting a specific goal. The first part aims to improve dynamic mobility, neuromuscular control and muscle strength. The second part includes small‐sided games to enhance technical skills, decision‐making and coordination, and the third part aims to develop explosive movements (Asgari et al. 2026a, 2026b). The poster and manual of the program are available at http://dx.doi.org/10.17877/DE290R‐26479 and http://dx.doi.org/10.17877/DE290R‐26478.

In contrast, the 11+ primarily focuses on controlled neuromuscular exercises, balance, core stability, and movement quality, with less focus on performance‐oriented and football‐specific loading characteristics. The 11+ program was described in detail by (Soligard et al. 2008).

At baseline, the health profiles, demographic characteristics, and motor performance metrics of all players were documented. The 12‐week intervention period began in week four of the preparation season and extended to week 10 of the competitive season. Each team performed its designated warm‐up routine twice weekly under the supervision of two research assistants (expert athletic trainers) blinded to the study design. Close collaboration with the coaching staff was maintained throughout the intervention period via regular communication from the lead researcher and on‐site support from the research assistants. The control group continued its standard warm‐up routines, including no specific injury prevention approach.

### 2.5 Test Battery

A standardized test battery was administered before and immediately after the 12‐week intervention period, and 10 weeks post‐intervention to evaluate the effects of the programs on motor performance and the retention of potential performance effects. The test battery included the linear 20‐m sprint (Arede et al. 2021; Zarei et al. 2018), the countermovement jump (CMJ) (Impellizzeri et al. 2013; Zarei et al. 2018), the Illinois agility (IA) (Hachana et al. 2014; Zarei et al. 2018), and the dribbling speed (DS) test (Höner and Roth 2015). The testing protocol followed the method outlined by Asgari et al. 2023. Performance changes between baseline and post‐intervention were analyzed to determine the effects of the programs, while differences between post‐intervention and the washout phase were examined to evaluate the retention of potential performance gains. Due to logistical constraints, one of the teams assigned to the Football+ group was unable to participate in the follow‐up assessment. Consequently, retention analyses were conducted using data from two teams, one from each intervention group.

All tests were conducted in a self‐command mode, with double‐light timing gates used to ensure precise measurements for time‐dependent tests, including linear sprinting, IA, and DS. Jump height was assessed using flight time measurements recorded with the OptojumpNext System (Microgate, Bolzano, Italy) (Castagna et al. 2013). To eliminate the acute effects of the programs on motor performance shown by (Asgari et al. 2023), all teams performed a standardized 10‐min warm‐up consisting of jogging and basic football drills prior to the measurements. Testing was conducted under consistent conditions, including comparable times of day (evenings), and weather (cloudy, 20°C–23°C in July, 10°C–12°C in October, and 3°C–5°C in December), and 50–70% humidity. All tests were performed on artificial turf, the same surface where the teams conducted their routine training (Figure 1).

**FIGURE 1:**
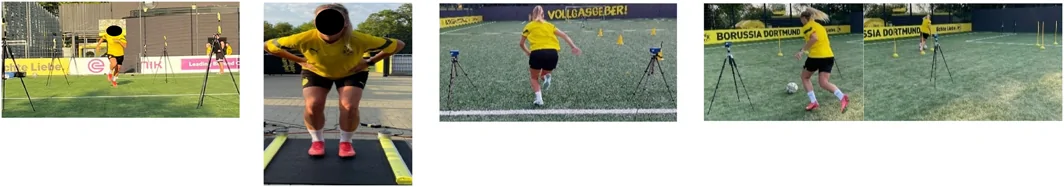
Motor performance test battery: linear sprint test, CMJ, IA, DS.

### 2.6 Statistical Analysis

All statistical analyses were performed using SPSS version 31 (IBM Corp., Armonk, NY, USA). Data were initially screened for normality using the Shapiro–Wilk test and for homogeneity of variance using Levene's test. Model‐based results are presented as estimated marginal means (EMMs) ± standard errors (SEs). Linear mixed‐effects models (LMMs) were applied separately to each dependent variable to account for the clustered team allocation and repeated measurements within players. For the overall analyses, time, group, and their interaction (group × time) were specified as fixed effects. Team nested within group [team(group)] was included to account for potential clustering associated with the team‐based allocation, while repeated measurements within participants were modeled to account for within‐subject dependency. Restricted maximum likelihood (REML) estimation was used, allowing all available observations to contribute to the analyses despite incomplete follow‐up data.

The group × time interaction constituted the primary effect of interest and was used to determine whether changes in motor performance differed between groups over time. Model‐based EMMs with corresponding SEs were obtained for each group and assessment time point. Absolute changes (Δ values) were calculated as differences between the respective model‐based estimates for the intervention period (post–pre) and, where available, the follow‐up period (follow‐up − post).

To directly quantify between‐group differences in changes during the intervention period, additional pairwise LMMs restricted to the pre and post‐assessments were conducted for the Football+ versus the 11+, the Football+ versus Control, and the 11+ versus Control. For each comparison, the estimated difference in change between groups (difference‐in‐differences; ΔΔ) was obtained from the group × time interaction and is reported with its 95% confidence interval (CI) and corresponding *p*‐value. Where applicable, Bonferroni‐adjusted pairwise comparisons were used to further examine significant interaction effects.

Standardized effect sizes for between‐group differences in pre‐post change were calculated as Hedges' g, applying a small‐sample correction. Effect sizes were derived from individual observed pre‐post change scores using the pooled standard deviation. The direction of the effect reflects the order of the groups included in each comparison and the direction of change in the respective performance outcomes. Statistical inference regarding between‐group differences was based primarily on the model‐derived contrast estimates, their 95% CIs, and corresponding *p*‐values. Because follow‐up measurements were unavailable for the Control group and one Football+ team, analyses involving follow‐up data were interpreted cautiously and considered exploratory with respect to the retention of intervention effects. All statistical tests were two‐sided, and significance was set at *p* < 0.05.

## 3 Results

### 3.1 Motor Performance Responses to IPPs at 12‐Week Post Test and Follow‐Up

Significant group × time interactions were observed for all performance outcomes, including 20‐m sprint (*p* < 0.001), CMJ (*p* = 0.013), Illinois agility (*p* < 0.001), and dribbling speed (*p* < 0.001; Table 2). Pairwise pre‐post analyses showed that the Football+ program resulted in significantly greater improvements than the 11+ in all four performance outcomes. Compared with the Control group, the Football+ also demonstrated significantly greater improvements in 20‐m sprint performance, CMJ height, and Illinois agility, whereas the difference in dribbling performance did not reach statistical significance. No significant differences in pre‐post changes were observed between the 11+ and Control groups for any outcome.

**TABLE 2:** Model‐based estimated marginal means, changes over time, and pairwise Pre‐Post contrasts.

Variable	Group	Pre (EMM ± SE)	Post (EMM ± SE)	Follow‐up (EMM ± SE)	Δ(Post‐pre)	Δ(FU‐post)	Group × Time p	Team (group) *p*	Football+ versus 11+ ΔΔ (95% CI); g; *p*	Football+ versus Control ΔΔ (95% CI); g; *p*	11+ versus Control ΔΔ (95% CI); g; *p*
20‐m sprint (s)	Football+	3.51 ± 0.02	3.38 ± 0.02	3.49 ± 0.03	−0.13	+0.11	< 0.001	0.352	0.08 (0.01–0.15); −0.69; 0.022	0.11 (0.06–0.15); −1.22; < 0.001	0.03 (−0.040–0.09); −0.23 0.43
	11+	3.48 ± 0.03	3.43 ± 0.05	3.43 ± 0.03	−0.04	−0.02					
	Control	3.46 ± 0.02	3.44 ± 0.04	—	−0.02	NA					
CMJ (cm)	Football+	25.70 ± 0.60	28.23 ± 0.56	26.95 ± 0.64	+2.53	−1.29	0.013	0.225	1.32 (0.07–2.58); 0.52 0.039	1.71 (0.69–2.73); 0.81; 0.001	0.39 (−0.95–1.73); 0.22 0.56
	11+	26.75 ± 0.74	28.18 ± 0.70	28.37 ± 0.74	+1.42	+0.19					
	Control	25.19 ± 0.61	26.06 ± 0.57	—	+0.88	—					
Illinois agility (s)	Football+	18.18 ± 0.10	17.28 ± 0.09	17.44 ± 0.11	−0.90	+0.16	< 0.001	0.853	0.81 (0.40–1.21); −1.13; < 0.001	0.75 (0.57–0.93); −1.88 < 0.001	−0.06 (−0.43 to 0.31); 0.09 0.75
	11+	17.89 ± 0.13	17.70 ± 0.12	17.50 ± 0.11	−0.19	−0.20					
	Control	17.91 ± 0.11	17.66 ± 0.10	—	−0.25	—					
Dribbling speed (s)	Football+	11.79 ± 0.14	11.52 ± 0.14	12.83 ± 0.19	−0.26	+1.30	< 0.001	< 0.001	0.72 (0.19–1.25); −0.95; 0.008	0.31 (−0.04–0.67); −0.50 0.08	−0.41 (−0.88 to 0.06); 0.60 0.09
	11+	11.87 ± 0.17	12.36 ± 0.18	12.45 ± 0.20	+0.50	+0.08					
	Control	11.93 ± 0.14	11.96 ± 0.15	—	+0.03	—					

*Note:* Values are estimated marginal means (EMMs) ± standard errors (SEs) derived from linear mixed‐effects models. Δ values represent changes between model‐based estimated marginal means. Pairwise contrasts (ΔΔ) represent the estimated between‐group difference in Pre–Post change, with 95% confidence intervals, obtained from separate linear mixed‐effects models restricted to the respective two groups and Pre/Post measurements. Hedges' *g* was calculated from observed individual Pre–Post change scores using the pooled standard deviation and small‐sample correction. The sign of *g* reflects the direction of the difference according to the order of groups used in the effect‐size analysis; for time‐based outcomes, negative values indicate a greater reduction in time in the first‐named group when applicable. Statistical inference is based on the mixed‐model contrast estimates and corresponding *p*‐values. Team was nested within Group in all models.

Regarding follow‐up, the Football+ group showed partial regression toward baseline in all outcomes, whereas the 11+ group generally maintained or further improved its performance in CMJ and agility. Follow‐up data should, however, be interpreted cautiously because measurements were unavailable for the Control group and one Football+ team.

The team(group) effect was not significant for 20‐m sprint, CMJ, or agility performance, but was significant for dribbling performance, indicating variability between teams for this outcome. Full model‐based estimates, within‐group changes, pairwise between‐group contrasts, 95% confidence intervals, standardized effect sizes, and corresponding *p*‐values are presented in Table 2.

## 4 Discussion

This three‐armed non‐randomized controlled trial examined the long‐term effects of the Football+ and 11+ programs on motor performance and their potential retention following a no‐intervention period among adult amateur female players. The findings revealed that a 12‐week supervised training with the Football+ twice weekly results in superior improvements in sprinting, agility, dribbling speed and vertical jump compared to the 11+ and traditional warm‐ups, with large effect sizes. Importantly, the mixed‐effect analyses indicated that between‐team variability had a negligible influence on sprinting, jumping, and agility outcomes, suggesting that the observed intervention effects were largely independent of team‐specific characteristics. However, the significant team‐level variability observed for dribbling performance suggests that factors such as coaching practices, team training culture, or implementation differences may also have contributed to the observed effects and should be considered when interpreting this outcome.

When compared to the control group, the 11+ showed noticeable improvements in vertical jump performance only, while a potential reduction in dribbling speed was also observed in that group. Similarly, Zarei et al. (2018) reported no improvement in dribbling skill following one‐season application of the 11+ among youth male players (Zarei et al. 2018). Additionally, Daneshjoo et al. (2013) found no improvement in dribbling speed in youth male players following 8 weeks of application of the 11+ (Daneshjoo et al. 2013). Our previous study (2023) demonstrated the superiority of the Football+ in optimizing acute performance including agility, CMJ, sprinting, and DS compared to the 11+ (Asgari et al. 2023).

The fact that 11+ may not influence technical skills can be explained by the absence of football‐specific exercises in the program. In contrast, the observed improvements in dribbling speed following the implementation of the Football+ can be attributed to the football‐specific nature of the program and align with existing research highlighting the efficacy of small‐sided games in developing technical skills (Zois et al. 2015). Further, in amateur football where training exposure is limited, coaches often prioritize tactical organization, match preparation, and general conditioning, leaving relatively little time for repeated execution of basic technical skills such as dribbling under controlled conditions. The consistent repetition of football‐specific drills embedded within the Football+ across the 12‐week intervention may have increased players' exposure to technically oriented movement patterns beyond what is typically achieved during regular team training. This repeated exposure may partially explain the observed improvements in dribbling performance.

Meanwhile, the present findings should also be interpreted in the context of the participants' initial performance status. Compared with values reported in higher‐level female football populations, the players included in this study demonstrated relatively modest baseline sprinting and jumping performances. Such lower initial values may indicate a greater capacity for adaptation in response to structured neuromuscular and football‐specific training stimuli. Therefore, the magnitude of the observed improvements may partially reflect the amateur status and developmental level of the participants. Consequently, caution is warranted when generalizing these findings to elite or highly trained female football populations.

Nine heterogeneous studies including different populations have evaluated the effects of the 11+ on vertical or long jump performance, four of which reported significant improvements, and the rest demonstrated no significant developments following application of the 11+ (Asgari, Nazari, et al. 2022b). When focusing specifically on studies involving adult male players, the literature tends to indicate that the 11+ is ineffective in improving jump performance, which conflicts with our findings (Asgari, Nazari, et al. 2022b). One plausible explanation for this discrepancy is gender differences in sport, demonstrating that males generally outperform females across the majority of physical tasks, and therefore, they may require more intensive training methods for performance enhancement. As a result, the level of difficulty of the 11+ might be sufficient to challenge jump performance in females but not in males. On the other hand, the greater volume and intensity of plyometric and anaerobic exercises incorporated within the Football+ may have contributed to the superior improvements observed in sprinting, agility, and jumping performance (Abade et al. 2017; Thomas et al. 2009). The 11+ integrates a series of strengthening exercises, especially in the second part, with a particular focus on core and hip muscles. Although beneficial for IP, this static structure may not properly challenge the motor performance and technical skills, and may not prepare the athletes for the main body of training.

As the final preparation phase before high‐intensity activities, warm‐up modalities are supposed to target both IP and performance enhancement (Fradkin et al. 2010). In this context, an IPP designed as a warm‐up routine that effectively balances both objectives can increase its applicability, practical value and user buy‐in, particularly for amateur football. Since both programs are easy to operate and require a similar time commitment (22–25 minutes in practice‐ 18.5% of total training time in this study), the Football+ presents a time‐efficient option that enhances performance and aligns well with the training goals. Therefore, the Football+ may represent a practical and time‐efficient warm‐up alternative for amateur female football teams seeking to simultaneously address performance enhancement and IP objectives. Additional research is needed to explore the Football+ impacts on biomechanical and biofeedback measures, as well as injury incidences to further validate it's IP values. Along with other studies, our previous study (2022) also illustrated that modifying the 11+ by roughly intensive and more dynamic exercises leads to significant improvements in dribbling speed and agility among amateur male players compared to the original 11+ and warm‐up routines (Asgari, Alizadeh, et al. 2022a).

Notably, the mixed‐effects analyses further indicated that the performance improvements observed during the intervention period were not fully maintained during the follow‐up phase. Although follow‐up data were unavailable for the control group and one Football+ team, the available observations suggest that the gains achieved during the intervention diminished after cessation of structured exposure. In particular, the Football+ group demonstrated a tendency to return toward baseline performance levels, whereas the 11+ group exhibited comparatively smaller changes during the no‐intervention period. These findings should be interpreted cautiously because the incomplete follow‐up structure limits definitive conclusions regarding long‐term retention. Consistent with the pattern observed in our study, Lopes et al. (2019) observed that improvements achieved following the 11+ program were not maintained after a 10‐week period without intervention, although their findings specifically concerned balance and proprioception (Lopes et al. 2019). Nevertheless, the observed attenuation of performance gains following cessation of training is consistent with the classical principle of reversibility in training, stating that physiological benefits and adaptations gained from consistent training can gradually diminish or be completely lost if training ceases or sharply reduces (Neil‐Sztramko et al. 2019). Essentially, “use it or lose it” underscores continuous training is necessary to maintain fitness and performance levels, and insufficient training can lead to declines in motor performance and physical capacities (Neil‐Sztramko et al. 2019). Thus, continued engagement with the Football+ throughout the entire season is essential.

The 11+ represents a major milestone in IP strategies and has substantially shaped the field since its introduction. It has been widely implemented and extensively studied across diverse populations and settings, contributing to the current evidence base. However, with the continued evolution of knowledge in areas relevant to IP, there is growing interest in optimizing existing programs by incorporating additional training stimuli. In particular, the inclusion of more demanding neuromuscular and performance‐oriented components may offer further benefits, both in terms of injury risk reduction and physical performance development. Emerging evidence suggests that factors such as program intensity, exercise progression, and delivery strategies may influence the effectiveness of IPPs (Benjaminse and Verhagen 2021; Singh et al. 2021). The present findings further support the notion that the inclusion of more demanding neuromuscular and football‐specific training stimuli may enhance the performance‐related benefits of IPPs.

It should be emphasized that the present study did not assess actual injury incidence; therefore, the findings should not be interpreted as evidence that the Football+ reduces injuries. Although IP remains a central rationale underlying the program design, its effectiveness in reducing injury occurrence requires evaluation in adequately powered prospective studies. Anecdotally, the Football+ was positively received by participating players, and players from teams assigned to the 11+ program also expressed interest in performing the Football+. However, as program acceptability and adherence were not systematically assessed, no conclusions can be drawn regarding whether Football+ is more attractive or results in better adherence than the 11+. Future studies should therefore investigate injury incidence alongside adherence, acceptability, and implementation fidelity.

## 5 Conclusion

The Football+ program was associated with greater improvements in sprinting, jumping, agility, and dribbling performance than the 11+ and traditional warm‐up routines in amateur female football players. The findings indicate that a more demanding, football‐specific warm‐up is a useful strategy for improving sprinting, jumping, agility, and dribbling performance in this population. However, the follow‐up data suggest that these benefits may depend on continued application. The attenuation of performance gains during the follow‐up period suggests that continued exposure may be necessary to maintain these adaptations.

## 6 Limitations

Several limitations should be considered when interpreting the findings of this study. First, the intervention was implemented in only five teams across three study arms. Consequently, the number of clusters was relatively small, which limits the precision of cluster‐level estimates and warrants cautious interpretation of the intervention effects. Nevertheless, the mixed‐effects analyses revealed that the observed effects were not substantially influenced by team‐level variability, except for dribbling performance.

Second, the study employed a cluster‐allocated rather than a fully randomized design, with one team self‐selecting into the control condition due to practical constraints. Although baseline characteristics were comparable between groups and supervised implementation procedures were standardized, the possibility of residual selection bias cannot be completely excluded. Therefore, the findings should be interpreted as evidence of associations between the interventions and performance adaptations rather than definitive causal effects.

Finally, the follow‐up assessment was affected by incomplete data availability, as one Football+ team was unable to participate and the control group was not assessed after the intervention period. Although linear mixed‐effects modeling allowed inclusion of all available observations, the reduced follow‐up structure limits the strength of conclusions regarding the long‐term retention of training adaptations.

## Author Contributions

M.A. derived the study concept, developed the Football+ program performed the statistical analysis, and drafted the manuscript. M.H. reviewed and revised the manuscript and refined the methodology. B.T.,M.S.^1^, performed the interventions, data collection, and preparation. M.S.^2^ managed the intervention phase and performed the introductory workshops, T.J. supervised the project, reviewed, and refined the manuscript.

## Funding

The authors have nothing to report.

## Ethics Statement

This study received ethics approval from the Ethics committee of TU Dortmund University (GEKTUDO_2022_43).

## Consent

The authors have the same contribution, read, and approved the final manuscript.

## Conflicts of Interest

The authors declare no conflicts of interest.

## Data Availability

The data that support the findings of this study are available on request from the corresponding author. The data are not publicly available due to privacy or ethical restrictions.
